# The Prevalence of Tic Disorders in Poland: Regional and Sex Differences

**DOI:** 10.5334/tohm.1165

**Published:** 2026-04-13

**Authors:** Katarzyna Śmilowska, Natalia Szejko, Aleksander J. Owczarek, Kinga Tomczak, Daniel J. van Wamelen, Kirsten R. Müller-Vahl

**Affiliations:** 1 Department of Neurology, 5th Regional Hospital in Sosnowiec, Poland; 2 Research Center for Public Policy and Regulatory Governance, University of Silesia in Katowice, Poland; 3 Department of Psychiatry, Social Psychiatry and Psychotherapy, Hannover Medical School, Hannover, Germany; 4 Department of Bioethics, Medical University of Warsaw, Poland; 5 Department of Pathophysiology, Health Promotion and Obesity Management Unit, Medical University of Silesia, Katowice, Poland; 6 Tic Disorders and Tourette Syndrome Program, Department of Neurology, Boston Children’s Hospital, Harvard Medical School, Boston, MA, United States; 7 Department of Neuroimaging, Institute of Psychiatry, Psychology & Neuroscience, King’s College London, London, United Kingdom; 8 Parkinson’s Foundation Center of Excellence, King’s College Hospital NHS Foundation Trust, London, United Kingdom

**Keywords:** Tic disorders, Tourette syndrome, Epidemiology, Adult tics

## Abstract

**Background:**

Primary tic disorders, including provisional tic disorder, chronic tic disorders and Tourette syndrome (TS), are common childhood-onset neurodevelopmental conditions. Epidemiological data from Central and Eastern Europe remain limited. This nationwide registry-based study provides the first population-level overview of tic disorders in Poland over a 14-year period.

**Methods:**

We analysed anonymised National Health Fund data from 2010–2024, identifying individuals diagnosed with tic disorders (ICD-10 F95), including provisional tic disorder (F95.0), chronic motor or phonic tic disorder (F95.1), and TS (F95.2). Comorbid ADHD (F90) and OCD (F42) were examined. Prevalence was stratified by age, sex, and region. Age at first diagnosis and healthcare utilisation were assessed. Healthcare utilisation was defined as the number of inpatient and outpatient encounters in which tic disorders were recorded as either a primary or comorbid diagnosis.

**Results:**

Recorded prevalence increased across all tic disorders. Prevalence was higher in males than females (p < 0.001), with male-to-female ratios ranging from 2:1 for provisional tics to 5–6:1 for TS in children. First diagnoses peaked between 6–12 years, with females diagnosed later than males (p < 0.001). Marked regional variation was observed, with higher prevalence in southern and southeastern regions (p < 0.001). ADHD and OCD were common, particularly in TS, and were associated with increased healthcare utilisation (p < 0.001). While total visits increased, visits per patient declined.

**Conclusions:**

Tic disorder prevalence has risen in Poland, with persistent regional disparities, highlighting the need for improved specialist access, earlier recognition, and integrated management of comorbidities.

## Introduction

Primary tic disorders encompass a spectrum of conditions that vary in duration, complexity, and symptom profile. According to DSM-5-TR [1] and ICD-10 [2], tic disorders include: Provisional Tic Disorder (F95.0), characterised by motor and/or phonic tics lasting less than one year; Chronic Motor or Phonic Tic Disorder (F95.1), defined by either motor or phonic tics (but not both) persisting for more than one year; and Tourette Syndrome (TS) (F95.2), which requires the presence of at least two motor and one phonic tics for at least one year with onset in childhood. While these disorders share common clinical features and neurobiological underpinnings, they differ in severity, tic type, and long-term course, making it important to contextualise TS within the broader classification of tic disorders.

While once considered rare, epidemiological studies indicate that TS affects nearly 1% of the global population and occurs three to four times more frequently in males [3,4,5,6,7,8]. Although tics often diminish in severity with age, longitudinal studies indicate that for a subset of individuals, symptoms persist into adulthood, potentially leading to functional impairment and psychosocial challenges [10]. In addition to the core motor and phonic symptoms, individuals with TS frequently experience psychiatric comorbidities, including attention deficit/hyperactivity disorder (ADHD) and obsessive-compulsive disorder (OCD), both of which may exacerbate functional difficulties and complicate treatment [10]. Notably, epidemiological studies indicate that the prevalence of ADHD and OCD varies across geographical regions, suggesting that environmental, cultural, and healthcare-system factors may influence their detection and expression [11,12].

Despite advancements in clinical research, large-scale epidemiological studies examining the prevalence and healthcare utilisation related to TS and tic disorders at a national level in individual countries remain scarce [20,21,22]. Comprehensive, population-based data are essential for understanding the burden of these conditions and for informing healthcare policy, service planning, and access to specialised care [23].

In this study, we conducted the first nationwide analysis of the prevalence of tic disorders and healthcare utilisation in Poland using National Health Fund (NHF) data from 2010 to 2024. We aimed to estimate the prevalence of diagnosed tic disorders across age groups and sexes, examine regional variation, and analyse patterns of healthcare utilisation, including the co-occurrence of ADHD and OCD.

## Methods

The analyses described here were based on data obtained from the NHF database in Poland (https://www.nfz.gov.pl). Population size estimates were sourced from the Central Statistical Office of Poland (https://stat.gov.pl). Central Statistical Office provides official annual population estimates stratified by age, sex, and region, which were used as denominators for prevalence calculations. Denominators were defined as the mid-year resident population of Poland for each calendar year (2010–2024), stratified by age group, sex, and province, as reported by the Central Statistical Office. These population estimates include all residents, irrespective of individual healthcare utilisation in a given year.

In Poland, publicly funded healthcare coverage is compulsory for the majority of adults through employment, social insurance, or family-based entitlement, while all individuals younger than 18 years are eligible for publicly financed healthcare regardless of insurance status. Consequently, the NHF database captures healthcare utilisation for the vast majority of the Polish population, ensuring appropriate alignment between numerator and denominator definitions. The NHF is a public body funded through compulsory health insurance contributions and is responsible for financing healthcare services and reimbursing the cost of medicines in Poland. Consequently, the NHF database contains records for all patients who received healthcare services financed from public funds, including those related to diagnosis of tic disorders (ICD-10 category F95) and the most frequent co-existing psychiatric comorbidities of tic disorders, ADHD (F90), and OCD (F42). It does not include patients diagnosed and treated entirely within the private healthcare sector; however, given the structure of the Polish healthcare system, such cases are likely rare. Most individuals with private healthcare insurance (approximately 13% of the population) also utilise public healthcare services [24].

The NHF database uses unique, anonymised patient identifiers that allow linkage of multiple healthcare records belonging to the same individual within and across calendar years. For annual prevalence estimates, each individual was counted only once per calendar year, regardless of the number of visits or repeated diagnostic entries within that year. Thus, the numerator represents the number of unique patients with at least one recorded ICD-10 F95 diagnosis in a given year. Because the dataset was anonymised prior to extraction and did not permit direct verification of lifetime diagnostic status before 2010, we were unable to distinguish incident from prevalent cases across the entire lifespan. Therefore, the same individual could contribute to prevalence estimates in multiple consecutive years if they continued to receive care. Our estimates should thus be interpreted as annual treated prevalence rather than cumulative or lifetime prevalence.

The NHF database identifies patient geographical location in one of 16 provinces. When analysing for regional differences, we categorized the following provinces as “southern”: Małopolskie, Podkarpackie, Dolnośląskie, Śląskie. We categorized the following provinces as “northern”: Pomorskie, Warmińsko-Mazurskie, Kujawsko-Pomorskie; “western”: Lubuskie, Wielkopolskie, Zachodniopomorskie; and “eastern”: Lubelskie, Podlaskie, Świętokrzyskie, Mazowieckie. For this analysis, we chose to examine the period 2010–2024 to provide a comprehensive longitudinal perspective. All data provided by the NHF had already been anonymised in accordance with the Polish Act on the Protection of Personal Data.

We defined cases by the presence of relevant ICD-10 codes for provisional tic disorder (F95.0) and chronic tic disorders (F95.1: chronic motor or chronic phonic tic disorder and F95.2: TS) recorded as either a primary or secondary (comorbid) diagnosis for any patient who received healthcare services within a given calendar year. Case identification was performed separately for each calendar year, and individuals were included in that year’s numerator if at least one qualifying diagnostic code was recorded during that year. Altogether, 57,844 cases of provisional tic disorder, 62,903 cases of chronic motor or phonic tic disorder, 24,289 cases of TS without comorbidities, 6,194 cases of TS comorbid with ADHD, 2,594 cases of TS comorbid with OCD and 609 cases of TS comorbid with ADHD and OCD were identified.

The database does not distinguish between diagnoses made in primary, secondary, or tertiary care settings. However, this is unlikely to substantially affect observed patterns across time, sex, age groups, or regions, as the distribution of care pathways and referral practices in Poland has remained relatively stable over the study period.

The primary objective of our study was to estimate the prevalence of chronic tic disorders in Poland from 2010 to 2024, stratified by province sex, and age groups. As no data on gender identity was available, we did not include it in the analysis. We calculated prevalence by dividing the annual number of patients with relevant ICD-10 codes by the average population for that year, both nationally and by region [25].

Furthermore, we conducted additional analyses to examine the age at first recorded diagnosis of tic disorders, aggregated into the same age groups and stratified by sex and regions. Age at first recorded diagnosis was defined as the age at the earliest appearance of an ICD-10 F95 code in the NHF database during the study period (2010–2024). It should be noted that this reflects the first recorded diagnosis within the available observation window and may not correspond to the true clinical onset of symptoms.

To assess the healthcare burden associated with the tic disorders, we analysed the number of reported healthcare visits and consultations, distinguishing between outpatient and inpatient services. Records were included when tic disorders were listed as either a primary or comorbid diagnosis. Data were stratified by province, sex, and age group (<18 years vs ≥18 years) to identify demographic and regional patterns in service use. In parallel, we determined the total number of healthcare visits for any diagnosis among paediatric (<18 years) and adult (≥18 years) patients by sex and region, allowing comparison of tic-related service use relative to overall healthcare utilisation. In instances where the number of hospitalisations for tic disorders was fewer than five, we extracted either the exact count or, when available, the corresponding percentage relative to all hospitalisations in that demographic or regional category. This approach provided a more complete picture of both the distribution and intensity of healthcare engagement among individuals diagnosed with chronic tic disorders across Poland.

A secondary objective was to assess the co-occurrence of ADHD (F90) and OCD (F42) diagnoses among patients diagnosed with chronic tic disorders. We estimated the prevalence of these comorbidities, defined as the number of patients with ADHD and/or OCD recorded as either a primary or secondary diagnosis alongside a tic disorder diagnosis. Additionally, we derived sex-specific prevalence estimates and further stratified the analysis by age categories: under 18 years and 18 years or older. Similarly, we analysed the age at first diagnosis of ADHD or OCD comorbid with tic disorders, aggregated into the same age categories and broken down by sex and province. We also assessed the number of healthcare visits/consultations, both outpatient and inpatient level, recorded with ADHD and/or OCD as a primary or comorbid diagnosis in conjunction with the diagnosis of tic disorder, stratified accordingly.

## Statistical analysis

Data were summarised descriptively and presented as numbers (percentages), prevalence ratio (PR) per 100,000 subjects, or as the number of visits per subject. As the data obtained from the NHF database in Poland are restricted to providing information only when there were more than 5 subjects in a province, we did not calculate prevalence ratios or visit rates. Statistical analysis was performed using STATISTICA 13.0 PL (Tibco Software Inc, Palo Alto, USA), and R software (v 4.4.0; R Development Core Team (2008). R: A language and environment for statistical computing. R Foundation for Statistical Computing, Vienna, Austria).

## Results

### Prevalence of Provisional Tic Disorders (F95.0)

#### Prevalence of Provisional Tic Disorders in Children and Adolescents (<18 years)

**Figure 1:**
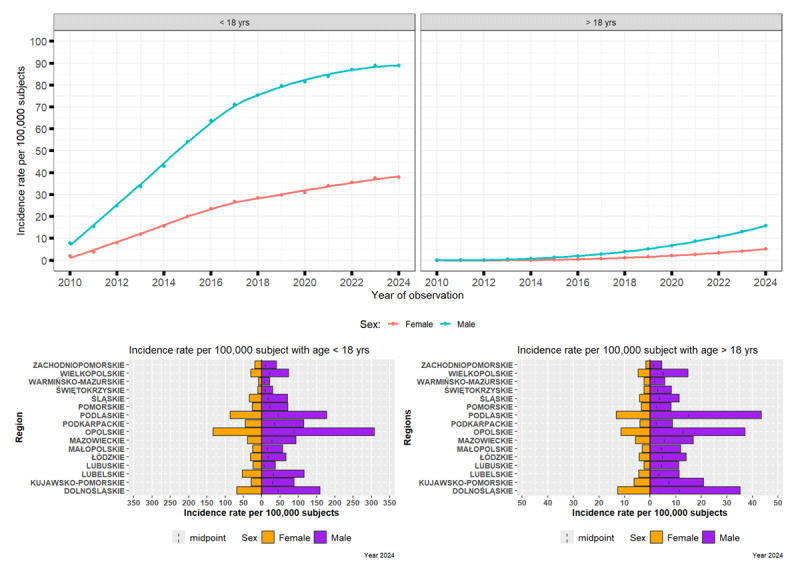
Evolving Patterns of Provisional Tic Disorders in Poland (2010–2024): Influence of Sex, Age, and Regional Factors.

Among individuals under 18 years of age, the prevalence of provisional tic disorders in Poland increased at each interval between 2010 and 2024. Prevalence was consistently higher in males than in females. Specifically, throughout the observation period, the prevalence in males was approximately three to four times greater. In 2010, the prevalence was 7.9 (95% CI: 7.0–8.9) per 100,000 in males and 1.9 (95% CI: 1.4–2.4) per 100,000 in females, increasing to about 88.9 (95% CI: 85.7–92.1) per 100,000 in males and 38.0 (95% CI: 35.9–40.1) per 100,000 in females by 2024 (Figure 1).

#### Prevalence of Provisional Tic Disorders in Adults (>18 years)

In the adult population (over 18 years), provisional tics were considerably less common but also demonstrated a gradual rise during the same period. In 2010, the prevalence was approximately 0.03 per 100,000 in both women and men. By 2024, these figures increased to 5.2 (95% CI: 4.9–6.0) per 100,000 in women and 15.7 (95% CI: 15.1–16.4) per 100,000 in men (Figure 1).

In the year 2024, the highest prevalence per 100,000 was observed in Opolskie (308.5) and Podlaskie (43.4) provinces in younger and older men, respectively, and in Opolskie (132.8) and Podlaskie (13.2) provinces in younger and older women, respectively. Of note, this diagnosis is only made in children and therefore these data probably correspond to misdiagnosis.

### Prevalence of Chronic Tic Disorders (F95.1 and F95.2)

#### Prevalence of Chronic Tic Disorders in Children and Adolescents (<18 years)

**Figure 2:**
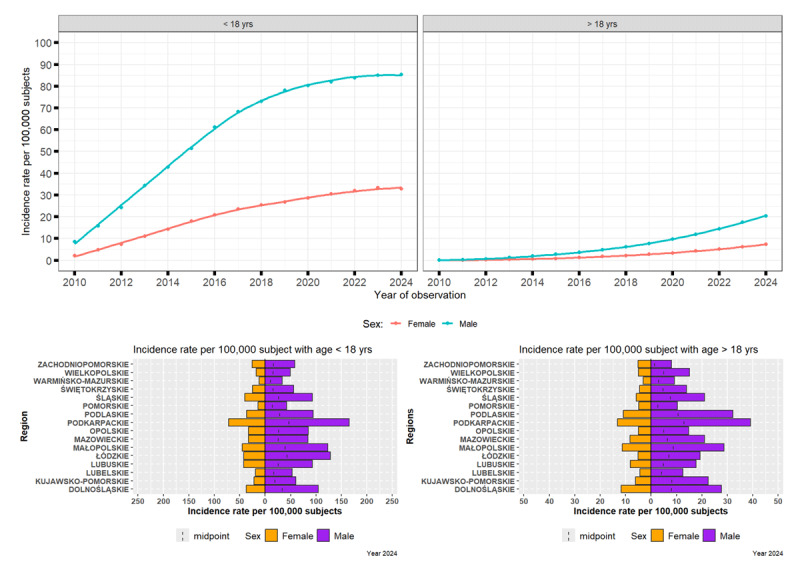
Evolving Patterns of Chronic Tic Disorders in Poland (2010–2024): Influence of Sex, Age, and Regional Factors.

Among individuals under 18 years of age, the prevalence of chronic tic disorders (motor or phonic) showed a clear increase at each interval between 2010 and 2024. The condition is significantly more common in males than in females. Throughout the observation period, prevalence in males was approximately three to four times higher. In 2010, the prevalence of chronic tics among males was 8.6 (95% CI: 7.6–9.6) per 100,000 compared to 2.1 (95% CI: 1.6–2.6) per 100,000 in females. By 2024, these figures had risen markedly to 85.4 (95% CI: 82.4–88.6) per 100,000 for males and 33.0 (95% CI: 31.0–34.9) per 100,000 for females (Figure 2).

#### Prevalence of Chronic Tic Disorders in Adults (>18 years)

Although prevalence in adults remained consistently lower than in children, increases were observed at each interval throughout the study period. In the adult population (over 18 years), chronic tics remained relatively rare but showed an increase across successive intervals. In 2010, prevalence was 0.13 (95% CI: 0.08–0.20) per 10,000 in men and 0.03 (95% CI: 0.01–0.07) per 10,000 in women. Over the following decade, increases in prevalence were observed across successive intervals, reaching 20.4 (95% CI: 19.7–21.2) per 10,000 in men and 7.3 (95% CI: 6.9–7.7) per 10,000 in women by 2024. Although the increase mirrored that seen in the paediatric population, the overall prevalence remained several times lower in adults (Figure 2).

In the year 2024, the highest prevalence per 100,000 was observed in Podkarpackie (165.2/39.1) province in younger and older men, respectively, and in Podkarpackie (71.5/13.2) province in younger and older women, respectively.

### Prevalence of Tourette syndrome

#### Prevalence of Tourette syndrome in Children and Adolescents (<18 years)

**Figure 3:**
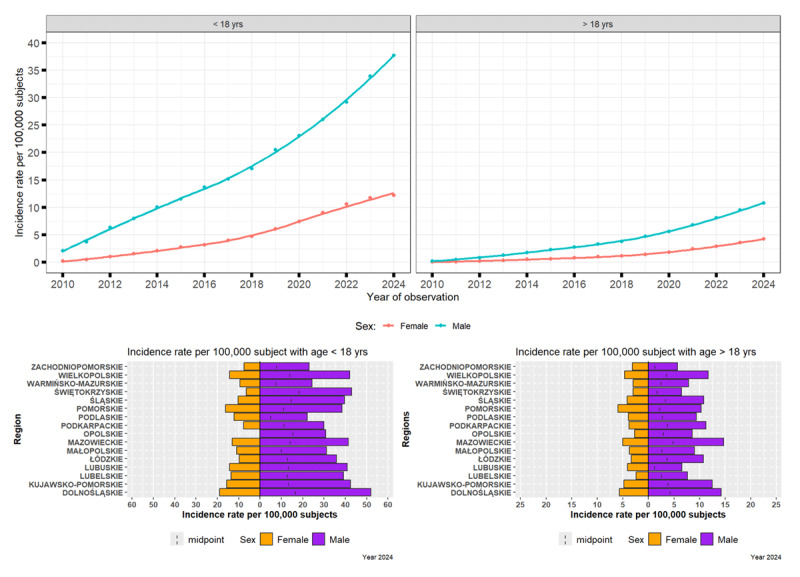
Evolving Patterns of Tourette syndrome in Poland (2010–2024): Influence of Sex, Age, and Regional Factors.

Among individuals under 18 years of age, the prevalence of TS in Poland increased across successive intervals between 2010 and 2024. The condition is significantly more common in boys than in girls, with male-to-female ratios remaining around 3:1 to 4:1 across the time period. In 2010, the prevalence was 2.1 (95% CI: 1.6–2.6) per 10,000 in boys and 0.14 (95% CI: 0.05–0.34) per 10,000 in girls. By 2024, these figures had risen sharply to 37.7 (95% CI: 35.7–37.7) per 10,000 in boys and 12.2 (95% CI: 11.0–13.4) per 10,000 in girls (Figure 3).

#### Prevalence of Tourette syndrome in adults (>18 years)

In contrast, the adult population shows a lower but progressively increasing prevalence over the same period. In 2010, prevalence was estimated at 0.20 (95% CI: 0.14–0.29) per 10,000 in men and 0.03 (95% CI: 0.01–0.07) per 10,000 in women. By 2024, prevalence had increased to 10.8 (95% CI: 10.2–11.3) per 10,000 in men and 4.2 (95% CI: 3.9–4.6) per 10,000 in women (Figure 3).

In the year 2024, the highest prevalence per 100,000 was observed in Dolnośląskie (52.0) and Mazowieckie (14.7) regions in younger and older men, respectively, and in Dolnośląskie (18.9) and Pomorskie (5.9) regions in younger and older women, respectively.

### Age at First Recorded Diagnosis

#### Provisional Tic Disorders

Provisional tic disorder was predominantly diagnosed in childhood, with the highest concentration of first diagnoses occurring between 6 and 9 years of age in both sexes. A substantial proportion was also identified during adolescence. Early onset before school age was observed more frequently among girls, whereas adult-onset cases were exceptionally rare across both sexes (Table 1).

**Table 1:** Distribution of age at first recorded diagnosis of tic disorders in Poland (2010–2024).


SEX	AGE CATEGORY	PROVISIONAL TIC DISORDER	CHRONIC TIC DISORDERS (PHONIC OR MOTOR)	TOURETTE SYNDROME

Female	0–3 yrs	1.7%	0.6%	0%

	4–5 yrs	8.9%	3.7%	1.7%

	6–9 yrs	40.5%	30.7%	13.7%

	10–18 yrs	44.3%	50.2%	51.3%

	>19 yrs	4.6%	14.8%	33.3%

Male	0–3 yrs	1.7%	0.7%	0.1%

	4–5 yrs	7.7%	3.6%	1.0%

	6–9 yrs	43.6%	34.5%	17.5%

	10–18 yrs	44.5%	53.8%	58.9%

	>19 yrs	2.5%	7.4%	22.5%


#### Chronic Tic Disorders (F95.1 and F95.2)

Chronic tics tended to present slightly later than provisional tics, with most first diagnoses made during adolescence. School-age onset remained common, while early childhood onset was infrequent. Adult diagnoses were relatively more frequent among women than men (Table 1).

#### Tourette Syndrome

TS exhibited the latest age at diagnosis among the three tic disorder categories, with the majority of cases identified during adolescence. Early onset in preschool or early school years was uncommon. Adult diagnoses were notably more frequent in women than in men (Table 1).

### Healthcare utilisation patterns for tic disorders

#### Patients under the age of 18

**Figure 4:**
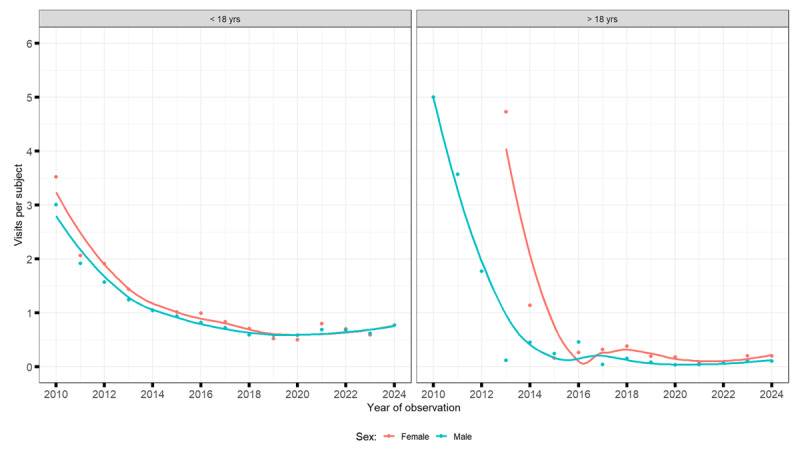
Number of visits for provisional tic disorders.

**Figure 5:**
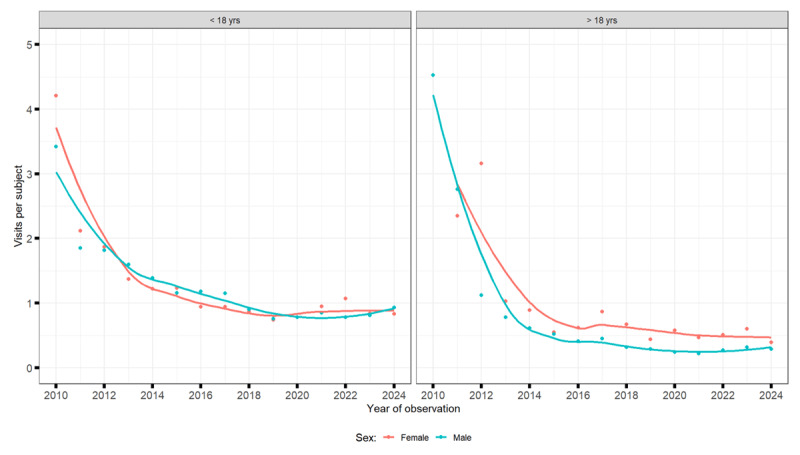
Number of visits for chronic tic disorders.

**Figure 6:**
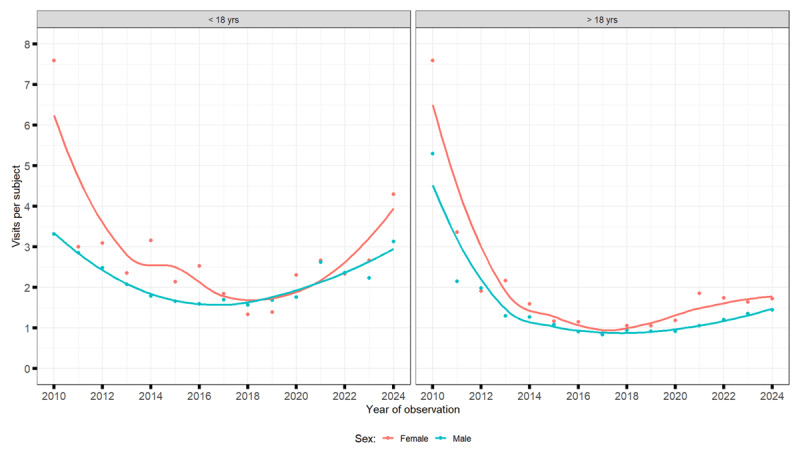
Number of visits in Tourette Syndrome.

Between 2010 and 2024, the total number of medical visits for tic disorders among children and adolescents increased sharply, alongside a growing number of patients. However, because patient numbers rose more rapidly, the average number of visits per patient decreased. Visits for transient tics grew from about 1,000 to over 3,000–4,000 annually, for chronic tics from under 2,000 to around 4,000, and for TS from a few hundred to over 4,000. Boys consistently accounted for about three times as many visits as girls (Figures 4, 5, 6).

#### Patients over 18 years of age

Among adults, both the number of visits and the number of patients increased, though the visits per patient declined slightly. Annual visits rose from fewer than 300 in 2010 to over 3,000 for chronic tic disorders, around 2,000 for TS, and several hundred for transient tics by 2024. Men made most visits, but the share of women increased over time, especially for TS and chronic tic disorders (Figures 4, 5, 6).

### Prevalence of comorbid adhd and ocd in patients with TS

Between 2010 and 2024, the prevalence of comorbid OCD and ADHD in patients with TS increased across all age groups and both sexes. The most pronounced rise was observed for ADHD in boys under 18 years (0.6 to 16.3 per 100,000), while OCD showed a more moderate but consistent increase (e.g., 0.2 to 2.5 per 100,000 in boys). Co-occurrence of TS with both ADHD and OCD remained rare but also increased over time. Most diagnoses occurred before age 18, particularly between 10 and 18 years (Table 3).

### Sex differences across diagnoses

Across all tic disorders, males consistently exhibited higher prevalence than females, though the magnitude of this disparity varied by disorder and age group (Table 2). The strongest male predominance was observed for TS, where the male-to-female ratio reached approximately 5–6:1 in children and around 6:1 in adults, confirming the well-established male bias in this type of tic disorder. Chronic (motor or phonic) tic disorders also showed substantial differences (about 3:1 in children and 2:1 in adults), while transient tic disorders presented a slightly smaller gap (around 2–2.5:1 in youth, narrowing further in adulthood).

**Table 2:** Male-to-female prevalence ratios by diagnosis and age group in Poland (in 2024).


DISORDER/DIAGNOSTIC CATEGORY	CHILDREN (<18 YEARS) (M:F)	ADULTS (≥18 YEARS) (M:F)

Provisional tic disorders	2.0–2.5 : 1	~1.5 : 1

Chronic tic disorders	~3 : 1	~2 : 1

TS	5–6 : 1	~6 : 1

TS + ADHD	5–6 : 1	~6 : 1

TS + OCD	2–3 : 1	~2 : 1

TS + ADHD + OCD	3–4 : 1	~3 : 1


**Table 3:** Prevalence of comorbid ADHD and OCD among patients with Tourette syndrome. Abbreviations: OCD: obsessive compulsive disorder; TS: Tourette syndrome; ADHD: attention deficit hyperactivity disorder.


COMORBIDITY	AGE GROUP	SEX	2010 PREVALENCE (PER 100,000)	2024 PREVALENCE (PER 100,000)

OCD + TS	<18 years	Male	0.2	2.5

	<18 years	Female	0.17	1.6

	≥18 years	Male	0.07	1.4

	≥18 years	Female	0.04	0.6

ADHD + TS	<18 years	Male	0.6	16.3

	<18 years	Female	0.2	2.9

	≥18 years	Male	0.07	2.6

	≥18 years	Female	0.04	0.4

TS + ADHD + OCD	<18 years	Male	0.14	1.1

	<18 years	Female	0.15	0.55


Comorbid conditions followed similar patterns: in TS with ADHD, the male-to-female ratio remained around 5–6:1 in children and 6:1 in adults, whereas TS with OCD showed more balanced but still male-dominant ratios of approximately 2–3:1 in childhood and 2:1 in adulthood. The combined TS + OCD + ADHD phenotype was rare but similarly male-predominant (roughly 3–4:1). Notably, sex disparities decreased with age across nearly all diagnoses.

## Discussion

Using national healthcare data, we have shown for the first time how tic disorder prevalence in Poland has changed over a fourteen-year period, revealing consistent increases in prevalence across all diagnostic categories, both sexes, and all age groups. The overall pattern aligns with global epidemiological observations, demonstrating a clear male predominance and a typical onset during middle childhood. Notably, substantial regional variation was observed, with the highest prevalence in the southern and southeastern regions (Małopolskie, Podkarpackie, and Dolnośląskie) and lower figures in the north and west of the country.

These record-based prevalence estimates were lower than survey-based point prevalence estimates of TS in the general population (~0.5–1%) [2,3,4,5,6,7]. This divergence, which is common in administrative studies, highlights the gap between community burden and the treated/recognised population captured by health systems, especially in girls and in regions with lower specialist density. Such datasets capture individuals who seek medical care and receive a formal diagnosis, rather than the full community prevalence. This difference likely reflects the gap between the true population burden and the treated or recognised population captured within healthcare systems.

Healthcare-related explanations could include differences in the availability of specialists, diagnostic infrastructure, and referral practices between provinces, potentially influencing detection rates rather than reflecting true underlying differences in prevalence [26,27]. Similar geographic gradients have been noted in other countries when care is concentrated in hubs [17,18,19,20]. Therefore, regional differences in administrative datasets should be interpreted cautiously and may primarily reflect patterns of healthcare access and diagnosis rather than true variation in disease occurrence. Importantly, regional differences observed in administrative data may reflect broader social determinants of healthcare utilisation, including access to specialists, educational awareness, and regional healthcare resource allocation [29]. Future research could explore this hypothesis by examining whether geographically similar patterns are present in unrelated paediatric or neurodevelopmental conditions; convergence of such patterns would support healthcare system–level drivers rather than biological variability.

In addition, recent years have seen increasing attention to functional tic-like behaviours and tic-related content disseminated through social media platforms. Exposure to highly visible tic-related material, particularly during adolescence, may contribute to increased symptom awareness, help-seeking behaviour, and diagnostic ascertainment. While this phenomenon does not imply a causal increase in neurodevelopmental tic disorders, it may partially influence recorded prevalence in administrative datasets through heightened recognition and referral [28].

Consistent with prior work, ADHD and OCD were common among individuals with tic disorders and showed distinct developmental timing—ADHD peaking earlier, OCD later into adolescence/young adulthood [1,9,10,11,12,13,14,15,16]. Comorbidity was associated with greater outpatient utilization, underscoring the need for integrated pathways: routine ADHD/OCD screening in tic clinics, as well as access to behavioral therapies such as habit reversal training or comprehensive behavioral intervention for tics (HRT/CBIT) and cognitive behavioral therapy with exposure and response prevention (CBT/ERP), and school-based accommodations to address attention, compulsivity, and stigma-related impairment [9,10,11,12,13,14,15,16]. Given that hospitalizations were rare, strengthening stepped-care outpatient models is likely the most efficient route to improving outcomes.

### Limitations

This study has several important limitations. First, the analysis was based on registry data, which depend on accurate reporting and coding. Regional variation in healthcare infrastructure, clinician expertise, and diagnostic practices may have influenced the observed incidence, potentially leading to underreporting in some areas and overestimation in others. In particular, it is possible that some of the patients categorized as having tics had, in fact, functional tic-like behaviours (FTLB) or other movement disorders such as stereotypies, which are also common in children. This is indicated, for example, by the fact that according to our data, there have been some adults diagnosed with the provisional tic disorder which probably indicates a misdiagnosis as this diagnosis is only made in children [30,31]. Second, while the dataset allowed for a comprehensive, nationwide assessment, it did not include individual-level clinical details such as symptom severity, age at onset, or presence of comorbidities beyond the diagnostic coding. As a result, the findings cannot fully capture the heterogeneity of clinical presentations or the functional impact of tic disorders. Third, sex differences, particularly the markedly lower incidence in females, should be interpreted with caution. These patterns may reflect underdiagnosis, sociocultural barriers to seeking care, or differences in symptom presentation, rather than true biological disparities. Finally, the study design was retrospective and descriptive, limiting causal interpretation. Increases in incidence over time may partly reflect greater clinical awareness, expanded access to healthcare, and improved documentation, rather than a true rise in disease occurrence. Nonetheless, we feel the findings presented here are relevant and reflect the real-world situation currently present in Poland.

## Conclusions

In conclusion, we observed (i) an increase in the recorded prevalence of all tic disorder categories between 2010 and 2024 across sexes and age groups; (ii) a persistent male predominance; and (iii) pronounced regional variation, with substantially higher prevalence in southern/southeastern province and lower in the north and west. From this, three immediate implications arise and based on these several suggestions can be made. First, implementation of standardised, sex-sensitive screening and referral prompts in paediatrics and schools to reduce female under-recognition. Second, levelling up of regional capacity, particularly in northern/western regions, through teleconsultation networks, outreach clinics, and dissemination of behavioural interventions (e.g., CBIT/HRT). Third, formalisation of integrated care with routine ADHD/OCD screening and brief CBT pathways, as comorbidity appears to be a major driver of service use and impairment.
